# Compressed Sensing Based Fingerprint Identification for Wireless Transmitters

**DOI:** 10.1155/2014/473178

**Published:** 2014-04-29

**Authors:** Caidan Zhao, Xiongpeng Wu, Lianfen Huang, Yan Yao, Yao-Chung Chang

**Affiliations:** ^1^Department of Communication Engineering, Xiamen University, Xiamen, Fujian 361005, China; ^2^Department of Computer Science and Information Engineering, National Taitung University, Taitung 95092, Taiwan

## Abstract

Most of the existing fingerprint identification techniques are unable to distinguish different wireless transmitters, whose emitted signals are highly attenuated, long-distance propagating, and of strong similarity to their transient waveforms. Therefore, this paper proposes a new method to identify different wireless transmitters based on compressed sensing. A data acquisition system is designed to capture the wireless transmitter signals. Complex analytical wavelet transform is used to obtain the envelope of the transient signal, and the corresponding features are extracted by using the compressed sensing theory. Feature selection utilizing minimum redundancy maximum relevance (mRMR) is employed to obtain the optimal feature subsets for identification. The results show that the proposed method is more efficient for the identification of wireless transmitters with similar transient waveforms.

## 1. Introduction

Fingerprint identification for wireless transmitters [1–3] is of great significance in safe radio communications [4–7], military communications confrontation, civilian radio monitoring, and so forth [8–11]. Owing to the discreteness of the wireless transmitter devices and inconsistent manufacturing levels [12, 13], the inherent fingerprint features can be extracted from the emitted signals. Hence, different transmitters can be identified from the captured signals in a fast way [14, 15].

The key process of individual identification is to extract the unique signal features that form a valid device fingerprint. The fingerprint of a transmitter should distinctly characterize it from the rest of the transmitters through its unique features presented in the signal waveform. The transient behavior varies from different transmitters due to imperfections in the analog components. Additionally, some wireless transmitter signals, such as the WiFi signal, are sent by frame under the conflict avoidance protocol, which facilitates transient signal acquisition. Therefore, the unique features extracted from transient signals can make a distinction among different transmitters by adopting the intercepted communication signals [16].

The transient feature extraction mostly utilizes the wavelet analysis or envelope analysis in the time-domain. Choe et al. have used the orthogonal Daubechies-4 wavelet to extract the transient signal features which are applied to identify two types of wireless transmitters at a recognition rate of 94.3% [17]. In the literature [18], wavelet coefficients are selected by genetic algorithms, and the rate of identification for communication stations is more than 90%. Statistical features are extracted from the energy envelopes of seven different manufacturers' Bluetooth devices by Rehman et al., and the average recognition rate reaches up to 99.9% [19]. In the literature [20], the energy envelope, obtained from the instantaneous signal by spectrogram analysis, is polynomial fitted to get the fitting coefficient characteristics. The result illustrates that these coefficient characteristics can effectively distinguish four different network cards. Zhao et al. have utilized complex analytic wavelet transform to obtain the envelopes from the signals and extracted the fitting coefficient characteristics using the Gaussian fitting method. The identification rate is up to 93% [21]. However, the characteristics of wavelet coefficients extracted by the wavelet analysis methods are of little difference and with large number. It requires high recognition performance of the recognizer. In addition, the above mentioned methods result in large differences of the signal waveforms, and most of their applications are for the short-distance WiFi signals and Bluetooth signals.

The compressed sensing theory can make the dimension of signals lower and all the information of original signal cannot be lost [22], and, therefore, this paper proposes a new method to identify different wireless transmitters based on compressed sensing to deal with the identification signals of high attenuation, long-distance transmission, and high transient waveform similarity. Experiments have been carried out in an outdoor environment. The distance between the transceivers is more than 3 km. Complex analytical wavelet transform is used to obtain the envelope of the transient signal, and features are extracted from the envelope based on the compressed sensing theory. A feature selection utilizing minimum redundancy maximum relevance (mRMR) is adopted to obtain optimal feature subsets for identification. The proposed method has also been compared to the techniques reported in the literatures.

The rest of this paper is organized as follows. In Section 2, signals acquisition is introduced. Furthermore, transient, envelope, and feature extraction of wireless transmitters is explained.  Section 3 gives an overview of analysis of recognition results and draws a comparison between the proposed technique and the reported techniques. Finally, the conclusion follows.

## 2. Methodology

**Figure 1:**
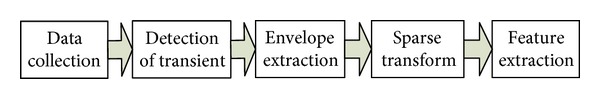
The flowchart of feature extraction for wireless transmitters.

The key step of fingerprint identification for the wireless transmitter is to extract unique features from the signals that different transmitters emit. To use compressed sensing theory, the sparseness of the signal has to be satisfied. However, lots of high frequency coefficients under the sparse-domain exist in a high-frequency signal, such as the wireless signal. Besides, sparse transform from a transmitter signal envelope can greatly increase the sparseness and it is convenient to extract the features using the compressed sensing. The proposed technique of features extraction is shown in Figure 1. Firstly, the envelope is extracted from the signals of starting-point detected according to the complex analytic wavelet transform. Then, the features are obtained from the envelope by utilizing the compressed sensing theory.

### 2.1. Data Collection

**Figure 2:**
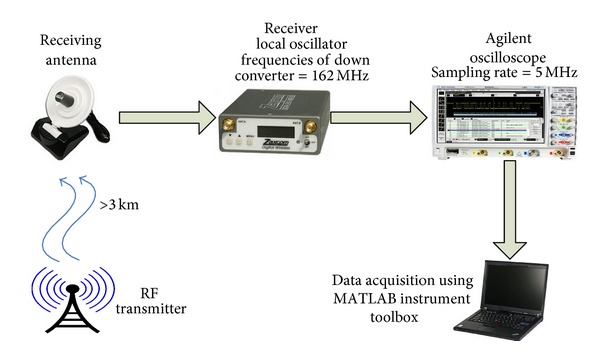
The block diagram of the signal acquisition for wireless transmitters.

**Figure 3:**
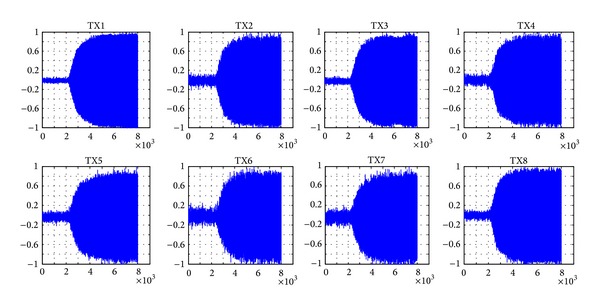
The transient waveforms of 8 different wireless transmitters.

Figure 2 shows the signal acquisition diagram. The sampling frequency is set as 5 MHz. The distance between the transmitting antenna and the receiving antenna is over 3 km. After being down-converted by the receiver, the intermediate-frequency signal is collected by an oscilloscope. The MATLAB instrument toolbox is used for transient and feature extraction. Due to the environmental impact, the receiving SNR of signals is around 10 dB to 20 dB. The transient signal waveforms of 8 different wireless transmitters, which are used for recognition analysis, are actually acquired as shown in Figure 3. The figure shows that there is no substantial difference among these waveforms. A total amount of 200 waveforms were captured and stored from each wireless transmitter during the device discovery mode.

### 2.2. Transient Extraction

Accurate transient detection is the key step to extract the features. Huang et al. have proposed a method of mean change point detection [23] to analyze the transient based on phase detection [24]. Its idea is to magnify the difference between the statistic of samples before and after the section, and the position of the maximum difference is determined to be the start of transient. The principle is as follows.

The sample sequence *x*_1_,  *x*_2_,…,  *x*_*N*_ is known (*x*_*i*_ corresponds to the phase variance in the method of phase detection).

Firstly, let *i* = 2,  3,…*N*. The samples can be divided into two sections: *x*_1_,  *x*_2_,…,  *x*_*i*−1_ and *x*_*i*_,  *x*_*i*+1_,…,  *x*_*N*_ for each number of *i*; then we can calculate the mean and statistics of each section. Consider

(1) $$\begin{array}{c} S_{i}=\overset{i-1}{\underset{t=1}{\sum }}{\left(x_{t}-\bar{X_{i1}}\right)}^{2}+\overset{N}{\underset{t=i}{\sum }}{\left(x_{t}-\bar{X_{i2}}\right)}^{2}. \end{array}$$

Secondly, $\bar{X}$ and *S* are calculated, which represent the average and statistics of the original sample, respectively. Consider

(2) $$\begin{array}{c} S=\overset{N}{\underset{t=1}{\sum }}{\left(x_{t}-\bar{X}\right)}^{2}. \end{array}$$

Thirdly, the curve of *S* − *S*_*i*_ is plotted. The position of maximum of the curve would be the change point.

**Figure 4:**
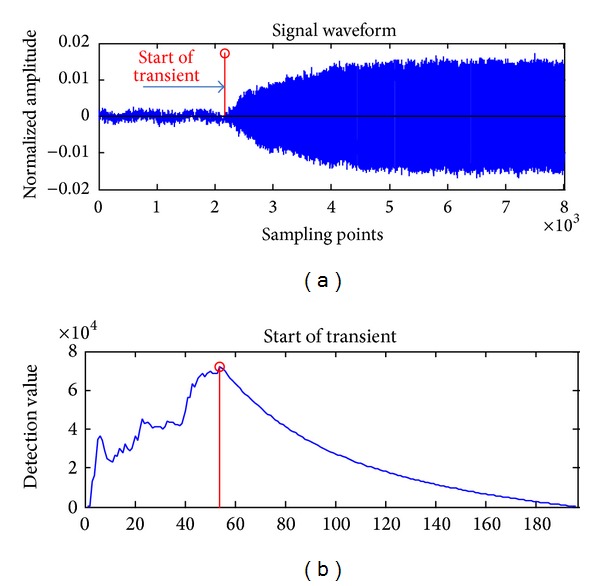
The detection for the start of wireless transmitter transient.

As shown in Figure 4, the detection for the start of wireless transmitter transient is accurate. Compared with the method of variance fractal dimension threshold detection [25], this method can increase the accuracy of detection without the limit of a threshold.

### 2.3. Envelope Extraction

**Figure 5:**
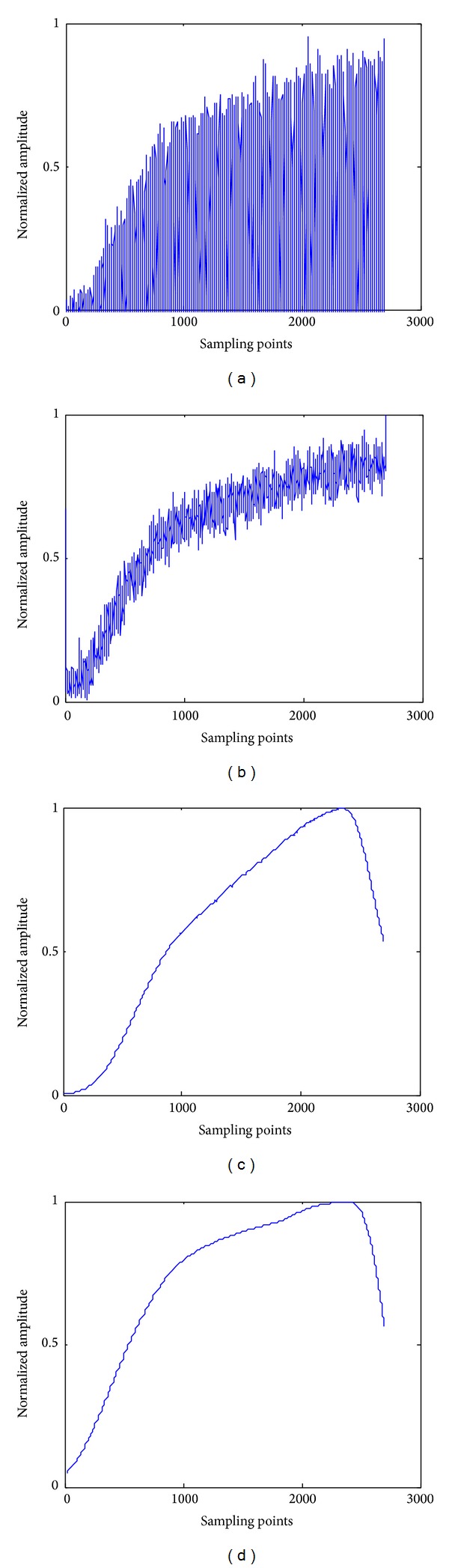
A comparison between Hilbert transform, spectrum slice analysis, and wavelet transform.

In a wireless communication environment, because of the interference of the background noise, signal envelope extracted by conventional Hilbert transform, which is shown in Figure 5(b), cannot effectively eliminate the effect of the random noise. It causes an adverse influence on the subsequent feature extraction [26]. In the literature [19, 20], the authors have proposed a method of spectrogram analysis to obtain transient envelope. However, due to the limitation in short time window, the transient envelope exhibits an obvious distortion during the extraction process. This extraction result is shown in Figure 5(c).

Recently, many scholars have put forward the method to extract the envelope from the signal based on complex analytical wavelet transform [26–28]. The envelope obtained by this method has an inhibited effect on random noise and shows a smooth envelope curve whose distortion is relatively petty.

The simplified expression of Morlet wavelet is as follows:

(3) $$\begin{array}{c} \phi \left(t\right)=\frac{1}{\sqrt{2\pi \sigma^{2}}}e^{-i2\pi f_{0}t}e^{-t^{2}/2\sigma^{2}},\;\sigma >0, \end{array}$$

where *σ* is the wavelet shape parameter (also known as bandwidth) and *f*_0_ is the wavelet center frequency.

For time-discrete signa *x*(*t*), *t* = *n*Δ*T*,  *n* = 1, 2,…, *N* − 1, where Δ*T* is the sampling period, Morlet wavelet transform of the signal can be expressed as follows:

(4) Wx(b,a)(m)=ΔTa2πσ2∑n=0N−1x(n)exp⁡[−(nΔT−b)22a2σ2] ×exp⁡(−i2πf0nΔT−ba),

where *b* = *m*Δ*T*,  *m* = 1, 2,…, *N* − 1, *a* is a scale factor. When *a* = *f*_0_/*f*_1_, *W*_*x*_ (*b*, *a*)(*m*) can get its maximum value. In the expression of *a* = *f*_0_/*f*_1_, *f*_0_ is the wavelet center frequency and *f*_1_ is the signal frequency. In order to ensure that the signal amplitude is equal before and after transformation, the envelope amplitude of the signal is 2|W_*x*_(*b*, *a*)(*m*)|.

To obtain a smooth envelope curve, the parameter *σ* and *f*_0_ of Morlet mother wavelet is adjusted. After lots of experiments, as shown in Figure 5(d), when *σ* = 7 and *f*_0_ = 1, the envelope curve is similar to the original signal and smoother.

**Figure 6:**
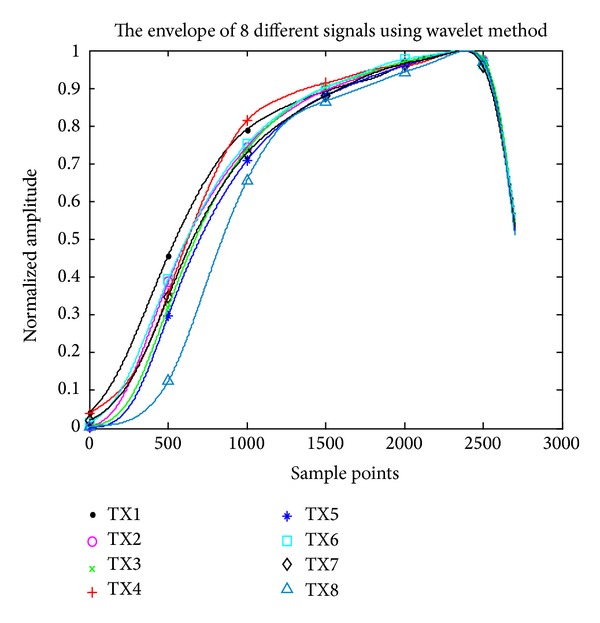
The envelope extraction of 8 different wireless transmitters using complex analytical wavelet transform.

The envelope extraction of 8 different wireless transmitters using complex analytical wavelet transform is shown in Figure 6. It can be seen that the envelopes have some differences between each other.

### 2.4. Feature Extraction Based on Compressed Sensing

The goal of the feature extraction is to form a unique transmitter fingerprint, which distinctly characterizes a transmitter from all of the transmitters. The method of this paper is to extract a feature from the envelope based on compressed sensing.

An orthogonal (sparse) transformation such as DCT (discrete cosine transform) [29] and DWT (discrete wavelet transform) [30] is performed for the envelope signal.

Assuming the length of the envelope signal *y* is *N*, *y* is able to be represented by the linear combination of *ψ* = {*ψ*_1_, *ψ*_2_ ⋯ *ψ*_*N*_}. Consider

(5) $$\begin{array}{c} y=\overset{N}{\underset{n=1}{\sum }}\psi_{n}s_{n}=\psi s, \end{array}$$

where *ψ* is an *N* × *N* orthogonal matrix to form an orthogonal transformation and *s* is an *N* × 1 coefficient vector. When *s* has *K* (*K* ≪ *N*) nonzero coefficients, the signal *y* is sparse under the matrix *ψ*.

(1) Linear measurement is the key process of the compressed sensing theory. The signal *y* is mapped to a measurement vector *z* which is a lower dimensional data. Consider

(6) $$\begin{array}{c} z=\Phi y=\Phi \psi s, \end{array}$$

where *z* is an *M* × 1  (*M* < *N*) vector, namely, the extracted features and Φ is an *M* × *N* measurement matrix. There are four kinds of measurement matrices including Gauss matrix [22, 31], Bernoulli matrix [22, 31], Toeplitz matrix [32], and sparse matrix [33]. The measurement matrix is critical for feature extraction. It is required that the measurement matrix can make the dimension of feature vector *z* as low as possible and all information of original signal cannot be lost.

(2) The reconstruction algorithm is used to confirm whether the features contain all information of the original signal, that is to say, whether the measurement *z* can recover the original signal *y*. The most direct way to reconstruct the signal is optimization problems in *L*0 norm:

(7) min⁡ ||s||l0s.t. z=Φψs,

where *s* is the sparse coefficients vector for reconstruction. Since the optimal computational complexity for reconstructing the signal is very high, Bayesian algorithm (BCS) [30] is usually used to find the approximate solution under acceptable complexity. Finally, the reconstruction signal $\tilde{y}$ is solved by Formula [16].

**Figure 7:**
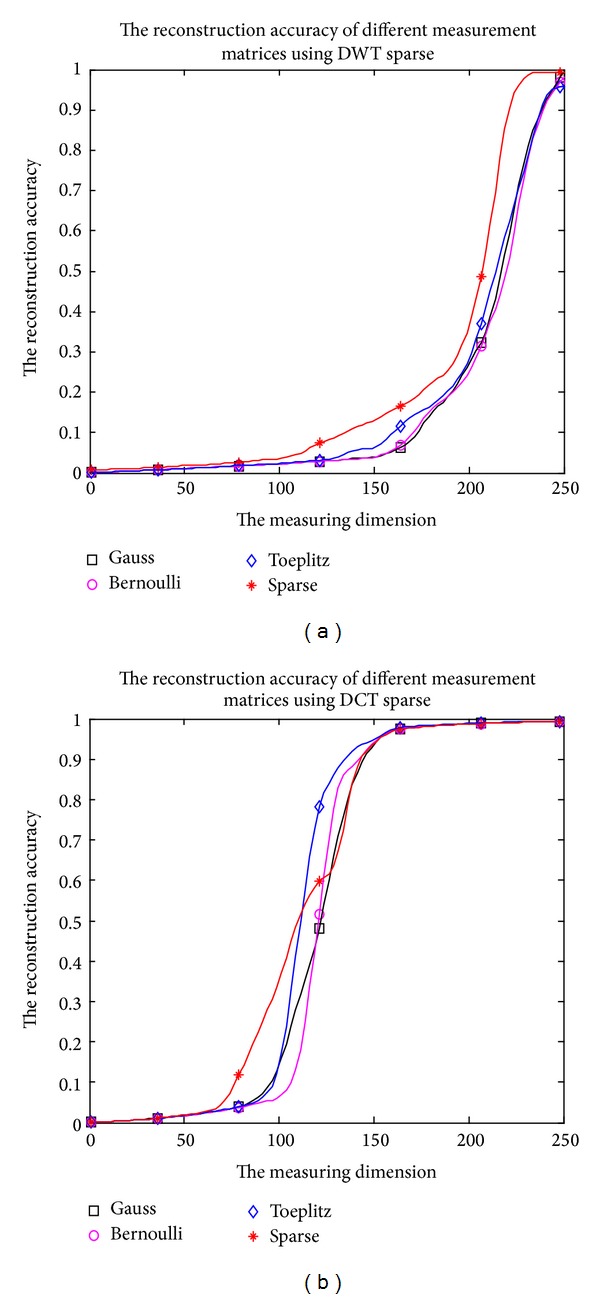
The reconstruction accuracy change with the measurement matrices and the dimension *M* of measurement vector *z*.

It is depicted in Figure 7 that the reconstruction accuracy changes with the measurement matrices and the dimension *M* of measurement vector *z*. It can be clearly shown that the sparse matrix is best suited for feature extraction. Taking into account the reconstruction accuracy, the dimension of the feature vector is set to 230 for DWT, while the dimension of feature vector is set to 160 for DCT.

(3) From the previous analysis, two feature vectors are extracted based on the DCT and DWT.

## 3. Recognition Results Analysis

In the stage of signal analysis, a recognizer combining multiclassification SVM recognizer [34] with BP neural network recognizer [35] is established. The “one-against-all” method needs more training time. And the appropriate parameters of multiclassification SVM are obtained by cross validation on the training data. To realize the multiclassification SVM, it is feasible to choose the “one-against-one” method, select the Gaussian radial basis function as Kernel, and set nuclear parameter and penalty factor as 1 and 1000, respectively. Furthermore, BP neural network recognizer is built by a three-layer neural network which contains only one hidden layer. The input layer neurons are set to be the number of dimensions of the transient fingerprint feature vector, and the output layer neurons are set to be the number of stations that need to be classified.

**Figure 8:**
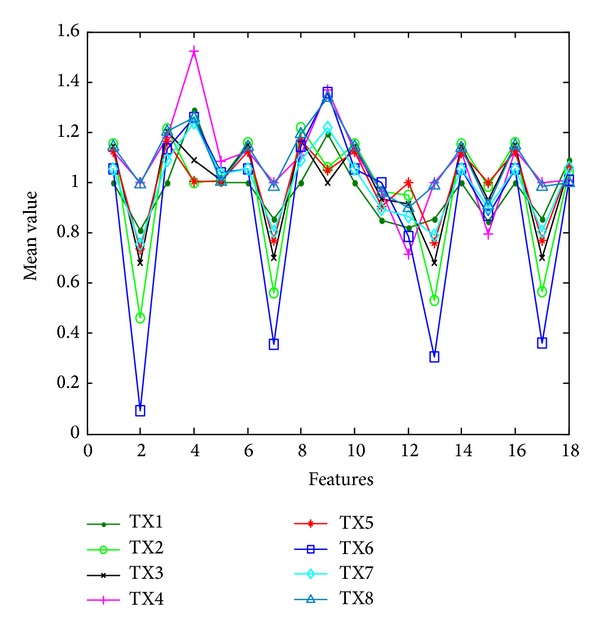
Averages of 18 features extracted from 100 groups of signals for 8 wireless transmitters.

As presented in this paper, the number of features extracted by the compressed sensing is too much, which results in a long time identification. The recognition rate decreases for some irrelevant feature interferences. Thus, the use of mRMR [36] to optimize the features is considerable. To directly analyze the effectiveness of the features we select, the distribution of 18 features' average values extracted from the 100 groups of signal of 8 wireless transmitters is shown in the Figure 8. It can be seen from the picture that the mRMR algorithm is an effective way to distinguish different wireless transmitters.

**Figure 9:**
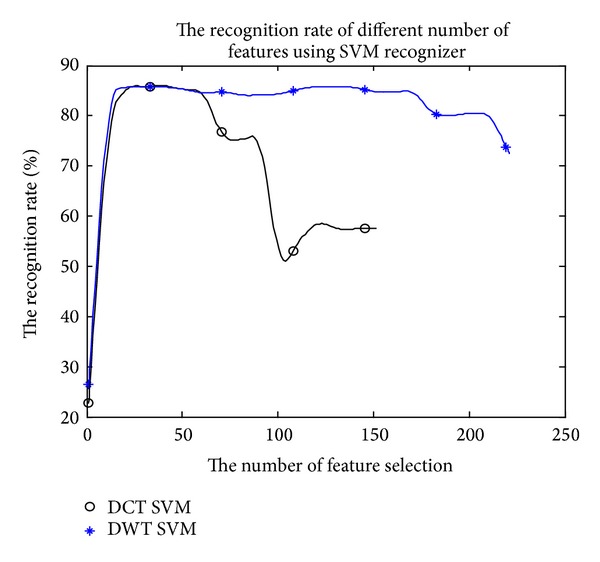
The comparison of the recognition results based on different sparse bases.

Figure 9 shows that the SVM identification results, which are through the envelope signal sparse by the DCT and DWT, with mRMR method to select features. The identification effectiveness is changing with the number of selecting features and different properties of sparse bases. In general, the convergence of DCT sparse base is better. If the number of the selecting features is under 50, the identification rate can be above 80%.

Through the envelope signal sparse by DCT sparse base, the SVM recognizer is utilized to identify 8 different wireless transmitters. For each transmitter, 100 groups are used for training and 100 groups for testing.  Table 1 presents the specific identification results. The data illustrate that the algorithm referred to in this paper is effective to identify transmitter when the average identification rates of most transmitters are over 88% and the average identification rate is 87.75%.

**Figure 10:**
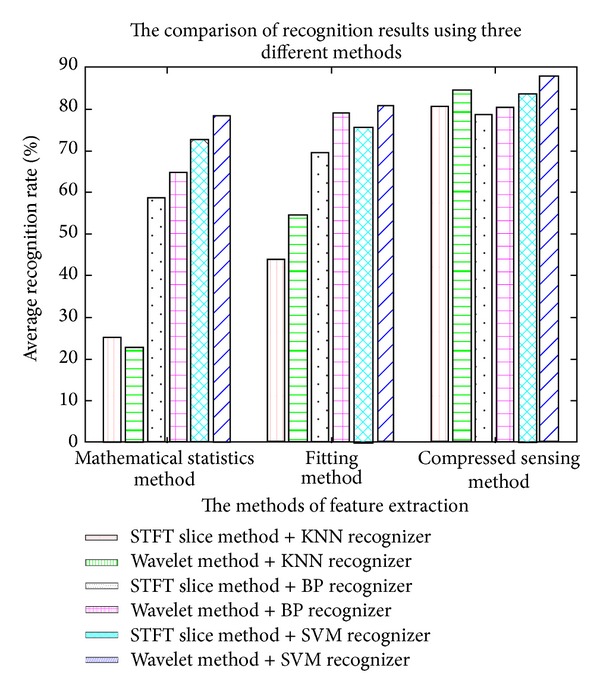
The comparison of classification results by three different methods.

The method proposed in this paper is compared with the works of Rehman et al. [19] and Zhao et al. [20, 21]. Rehman's work simply extracts the envelope statistic features from the energy spectrum envelope. And Zhao et al. put forward using the fitting coefficients of the envelope as the signal features for identification.  Table 2 describes the comparison among Reham's work, Zhao's work, and the proposed work in this paper. The term mathematical statistics method refers to Rehman et al.'s work, fitting method is used for Zhao et al.'s work, and compressed sensing method represents the work proposed in this paper. The 8 RF signal transmitting devices identified in this paper are similar in their transient waveforms. The recognition rates of either Rehman's or Zhao's method are relatively lower.  Figure 10 shows the specific comparison result. As shown in the figure, the extracted envelope features method based on compressive sensing theory in this paper has many advantages over the existing statistic features and fitting coefficient characteristics methods. On the one hand, extracting envelope by complex analytic wavelet is superior to spectrum slicing. On the other hand, the recognition performance of SVM recognizer is better than both BP neural network recognizer and KNN recognizer.

According to the disadvantages of the previous methods and the merits of our proposed algorithm, performance comparison for the envelope extraction and feature extraction is provided as Table 3.

## 4. Conclusions

This paper has proposed a fingerprint identification method for wireless transmitter signal based on compressed sensing. Complex analytical wavelet transform is used to obtain the envelope of the transient signal, and features are extracted from the envelope using the compressed sensing theory. A feature selection utilizing minimum redundancy maximum relevance (mRMR) is employed to obtain optimal feature subsets for identification. Finally, the recognition of 8 wireless transmitters by the SVM recognizer and BP neural network recognizer is completely performed. From a series of experiments, it can be concluded that the method proposed in the paper can effectively identify the transmitter signals, whose transient waveforms are difficult to distinguish with the naked eye. Especially, the method put forward in the paper has better performance in the recognition of wireless transmitter signals with serious interference and signal attenuation when compared to the previous methods, such as extracting statistic features directly from the envelope, fitting coefficient characteristic. What is more, this method may be applied to identify the signals which are transmitted by the transmitters with the same batch and type. According to the results, a future research on the optimization of the Morlet wavelet's parameter is expected to improve the performance of the proposed method.

## Figures and Tables

**Table 1:** Classification results for different wireless transmitters.

Input	Classification								Average rate (%)
	TX1	TX2	TX3	TX4	TX5	TX6	TX7	TX8	
TX1	**90**	0	2	0	0	3	5	0	90
TX2	1	**80**	6	1	5	7	0	0	80
TX3	1	9	**88**	1	0	1	0	0	88
TX4	3	0	0	**91**	0	2	0	4	91
TX5	0	5	1	0	**93**	0	0	1	93
TX6	3	2	2	5	3	**79**	3	3	79
TX7	5	0	2	3	0	2	**88**	0	88
TX8	1	0	1	2	1	0	2	**93**	93

**Table 2:** Comparison of identification of a transmitter using three different methods.

Comparison metric	Mathematical statistics method	Fitting method	Compressed sensing method
RF fingerprints	Area under the normalized curve, duration, maximum slope, kurtosis, skewness, and variance of curve	Gaussian fitting coefficients	Feature extracted as explained in subsection II
Envelope extraction	STFT slice method	Wavelet method STFT slice method	Wavelet method
Classifier	KNN	SVM and BP	SVM and BP

**Table 3:** Performance comparison for the envelope extraction and feature extraction.

	Envelope extraction		Feature extraction		
	STFT slice	Wavelet	Mathematical statistics	Fitting	Compressed sensing
Advantage	Eliminate the effect of the noise	Envelope is smoother and precise	Simple; the feature is easy to extract	Simple; the features are easy to extract	The features are of high representativeness
Disadvantage	Envelope exhibits a distortion	Parameters are determined by experience	Some features are not representative	Fitting function is determined by experience	Complex; need a long time
Complexity	*O*(*n* ^2^)	*O*(*n* ^2^)	*O*(*n*)	*O*(*n*)	*O*(*n* ^2^)
Time for calculation (s)	2.251245	3.568356	1.324417	2.419341	4.626052
